# 

**Published:** 1898-01-29

**Authors:** 


**Notices of Births, Deaths, and Marriages** are inserted in this column at a charge of 2s. 6d. for an announcement not exceeding four lines.

### NOTICE TO CORRESPONDENTS.

All MS., Letters, Books for Review, and other matters intended for the Editor should be addressed THE EDITOR, "THE HOSPITAL," THE HOSPITAL BUILDING, 28 & 29, SOUTHAMPTON STREET, STRAND, W.C.

The Editor cannot undertake to return rejected MS., even when accompanied by stamped directed envelope.

**Notice to Advertisers and Subscribers.**

All Advertisements, Orders for Copies of THE HOSPITAL, and Business Communications should be addressed to **THE MANAGER, (Not to The Editor).** THE HOSPITAL, The Hospital Building, 28 & 29, Southampton Street, Strand, London, W.C.

The Cost of Subscription, post free, is as follows:—Per week, 2½d. per quarter, 2s. 8d.; per half-year, 5s. 3d.; per annum, 10s. 6d. Foreign:— Per annum, 15s. 2d.: payable, in all cases, in advance.

**NOTICE.—Vol. XXII. of "The Hospital" (April, 1897, to September, 1897), in handsome cloth binding, is now on sale at the Office of this Journal, price 7s. 6d. Cases for binding the Numbers contained in this Volume. 1s. 6d. Index to Vol. XXII., 2½d., post free. The Monthly Part for February will be ready on February 1st. Price 6d., by post 8d.**

### SCRAPS AND GLEANINGS.

KLONDYKE is to have a typewriting office, a Remington having just been ordered for use there.

THE total insane population of the two asylums in the Central Provinces of India in 1896 was 382.

THE 16 hospitals and dispensaries in Calcutta between them provide accommodation for 1,835 patients.

THE Prince of Wales has sent a present of 20 pheasants to the patients of the Metropolitan Hospital, of which institution he is a patron.

CHOLERA was more fatal in 1896 in Calcutta than in any previous year since 1889, having caused 3,449 deaths against 2,955, the next largest, in 1891.

THE result of the fête held at Roundhay Park in connection with the Leeds Workpeople's Hospital Fund this year was not satisfactory. Only £245 was realised, against £604 in 1896.

AMONG female medical practitioners in the Panjab the largest number of operations performed in the dispensaries in that district were done by Miss Elizabeth Bielby, of the Lady Aitchison Hospital, Lahore.

THE Ely Dispensary Managers have decided that, unless the letter of recommendation be endorsed by the subscribers to the effect that the patient cannot afford to pay it, each patient be charged a fee of 1s.

THE Liverpool Hospital Sunday Fund collection shows a falling off in 1897 when compared with 1896 from £6,334 in the latter year to £6,003 in the former. The Saturday Fund collection increased from £5,225 in 1896 to £5,869 in 1897.

THE liability to the bank by the Birmingham Orthopædic Hospital reached £1,514 at the end of last year, and it was stated at the annual meeting that unless help was speedily received it would be necessary to close some of the wards.

EIGHT thousand and eighty-eight pounds was collected in 1897 by the Manchester Hospital Sunday and Saturday Fund, a decrease of £227 compared with 1896. The same amount, viz., £7,500, was, however, distributed in both years.

CLASSIFIED according to race, nationality, or religion, the 284,441 patients treated in the Calcutta medical institutions in 1896 may be divided as follows: Europeans, 7,657; Eurasians, 34,833; Mohammedans, 88,223; Hindus, 142,148; other classes, 11,580.

THE Earl de la Warr and Mr. Daniel Mayer have withdrawn their offers of contributions to the proposed Bexhill Cottage Hospital; the former because his conditions had not been fulfilled, and the latter because of the inadequate support given to the scheme.

THE average number of in-patients at the Glasgow Western Infirmary in 1896-7 was 386, a slight increase over the average for 1895-6, viz., 38[illegible]. The average residence of each patient was practically the same in both years. Altogether 4,548 in and 14,766 out-patients were treated.

THE council of Charing Cross Hospital have received a sum of £233, the result of the concert given on November 19th in aid of the Special Appeal Fund, by the Royal Amateur Orchestral Society, under the immediate patronage of the Duke of Saxe-Coburg and Gotha, president of the hospital.

THE ordinary expenditure during the last fiscal year at the Stourbridge Dispensary amounted to £354, for which sum 2,342 patients were treated. The patients showed an increase over the previous year of 693, due almost entirely to the fortnightly tickets issued for the first time early in the year. The income was £385.

THERE were 34 police hospitals, and 17 special wards for police, open in the Madras Presidency at the end of 189[illegible], making provision for 655 in-patients. Nine thousand six hundred and thirty-eight in, and 32,916 out-patients were treated during 1896, against 10,176 in and 34,453 out-patients in the previous year.

THERE is a steady tendency towards an increase in the number of medical institutions in the Panjab. The triennial period ended 1895 showed an increase of 15 dispensaries in that period, and at the end of 1896 there was a further increase of 10. The total number of institutions open on December 31st, 1896, was 267.

THE number of State and Local Fund dispensaries in the Central Provinces of India remained the same in 1896 as in 1895, viz., six of the former and 80 of the latter. There are also 83 private dispensaries. The total number of patients treated rose from 1,261,590 (12,224 in and 1,249,366 out-patients) in 1895, to 1,282,632 (13,193 in and 1,269,439 out-patients) in 1896.

THE committee of the Hull Hospital Sunday Fund are to be congratulated on the success of their endeavour during the year just passed to raise the Sunday collection to £1,000. The total reached no less than £1,134, and this was due in a great measure to the illustrated handbook called the "Souvenir of the Hull Medical Charities," issued by the committee to the householders of the city.

SLIGHTLY fewer patients were treated at the Plymouth Public Dispensary during the year just ended than in the previous year, the figures being 3,286 and 3,778 respectively. There was a deficit of about £138 at the end of the year, of which £100 had been met by taking that sum from capital. The deficit was stated to be the result of the recent increase in the number of tickets given to subscribers.

THE number of beds for patients provided in 18[illegible] in the various provinces of India per 1,000 of the population is as follows: Assam, 0·09; Bengal (exclusive of Calcutta), 0·04; Calcutta, 1·9; Bombay Presidency (exclusive of the town), 0·1; Bombay Town, 1·5; Burmah, 0·3; Central Provinces, 0·06; Hyderabad Assigned Districts (exclusive of Secunderabad), 0·1; Secunderabad, 0·9; Madras Presidency (exclusive of the town), 0·1; Madras Town, 2·9; North West Provinces and Oudh, 0·08; Panjab, 0·15.

THE secretary of the British Home for Incurables has received a donation of £5 14s. 11d. from Her Royal Highness the Princess of Wales, patroness of the charity, being a farther sum derived from the sale of Canon Fleming's sermon, "Recognition in Eternity," making a total of £702 5s. 11d. The secretary has also received the sum of £1,000 from "A Lady" for a bed in the home, to be known as the "James and Eliza Woodall" bed, and to which she has nominated James Rickards from the list of candidates.

### The Student of Medicine.

**APPOINTMENTS.**

J. W. Benson, L.R.C.P.I., L.R.C.S.I., L.M., appointed Resident Surgeon of the North Riding Infirmary, Middlesbrough-on-Tees. J. Clough, L.R.C.P.Lond., M.R.C.S.Eng., appointed Medical Officer for the First District of the Hunslet Union. W. S. Colman, M.B., C.M., appointed House Surgeon to the Edinburgh Royal Maternity and Simpson Memorial Hospital. Dr. Crawford, appointed Medical Officer for the Fifth District of the Tonbridge Union. H. J. Curtis, B.S.Lond., F.R.C.S.Eng., appointed Surgical Rrgistrar to University College Hospital, London. J. H. De Villiers, M.R.C.S., L.R.C.P.Lond., appointed House Physician to the Hospital for Consumption and Diseases of the Chest, Brompton. A. Dobson, M.R.C.S.Eng., &c., Surgeon to the Ilkeston Hospital; and Honorary Lecturer and Surgeon to the Ilkeston Ambulance Association. B. Dyball, M.B., B.S.Lond. (bracketted Gold Medal and Scholarship) F.R.C.S., L.R.C.P.Lond., appointed Resident Casualty Office to the General Infirmary, Leeds. C. W. Edwards, M.R.C.S., L.R.C.P., appointed Surgeon to the East Grinstead College Hospital. J. D. Holmes, M.B.Glas., appointed House Surgeon to the General Infirmary and Fever Hospital, Paisley. Dr. Jepp, appointed Medical Officer of Health to the Whitchurch Rural Council. R. H. Lucy, M.B.Edin., F.R.C.S.Eng., appointed Surgeon to the South Devon and East Cornwall Hospital, Plymouth. R. Maples, L.R.C.P.Edin., L.M., M.R.C.S.Eng., appointed Medical Officer of Health to the Kingsclere District Council. Dr. Moffat, appointed Medical Officer to the Alness Parish Council. P. A. Palmer, M.R.C.S.Eng., L.R.C.P.Lond., Junior Assistant House Surgeon, appointed Assistant House Surgeon of the Hull Royal Infirmary. H. J. Pickering, L.D.S.R.C.S.Eng., appointed Assistant Dental Surgeon to York Dispensary. E. E. Porritt, M.B., C.M., appointed House Surgeon to the Edinburgh Royal Maternity and Simpson Memorial Hospital. J. M. S. Preston, M.B., C.M.Edin., appointed Assistant Medical Officer of the New Bridge Street Workhouse of the Township of Manchester. H. A. Scott, M.B., Ch.B.Vict., M.R.C.S., L.R.C.P., appointed Third Assistant Medical Officer to the Monsall Fever Hospital, Manchester. H. C. Thomson, M.D., M.R.C.P.Lond., appointed Physician to Out patients at the Hospital for Epilepsy and Paralysis, Regent's Park. Dr. Tinker, appointed Medical Officer for the No. 7 (Newton) District of the Ashton Union. H. A. Ballance, M.S.Lond., F.R.C.S.Eng., appointed Assistant Surgeon to the Norfolk and Norwich Hospital. H. P. Bennett, M.B., C.M.Edin., appointed Assistant Surgeon to the Northumberland, Durham, and Newcastle Infirmary for Diseases of the Eye. M. Beverley, M.D.Edin., M.R.C.S.Eng., appointed Consulting Surgeon to the Norfolk and Norwich Hospital. S. H. Burton, M.B.Lond., B.S., F.R.C.S.Eng., appointed Surgeon to the Norfolk and Norwich Hospital. C. J. Caddick, M.B., C.M.Edin., appointed Assistant Medical Officer to the City Hospital, Edinburgh. T. G. Crewdson, M.B., C.M.Edin., appointed Assistant Medical Officer to the City Hospital, Edinburgh. C. S. Edwards, L.R.C.P.&S Edin., L.F.P.S.Glasg., appointed Certifying Surgeon under the Factory Act for the Tunstall District. H. N. Edwards, M.R.C.S., L.R.C.P., appointed Medical Officer for the Flamsted District of the Hemel Hempstead Union.

